# Temporal Dynamics of Adverse Effects across Five Sessions of Transcranial Direct Current Stimulation

**DOI:** 10.3390/brainsci14050457

**Published:** 2024-04-30

**Authors:** Miguel Delicado-Miralles, Laura Flix-Diez, Francisco Gurdiel-Álvarez, Enrique Velasco, María Galán-Calle, Sergio Lerma Lara

**Affiliations:** 1 Department of Pathology and Surgery, Center for Translational Research in Physiotherapy, Miguel Hernández University, Sant Joan d’Alacant, 03550 Alicante, Spain; mdelicado@umh.es; 2 Physiotherapy Faculty, Universidad de Valencia (UV), 46010 Valencia, Spain; lflix@unav.es; 3 Department of Physical Therapy, Occupational Therapy, Rehabilitation and Physical Medicine University of Rey Juan Carlos, 28922 Alcorcón, Spain; f.gurdiel.2019@alumnos.urjc.es; 4 Laboratory of Ion Channel Research, Department of Cellular and Molecular Medicine, KU Leuven, VIB-KU Leuven Center for Brain & Disease Research, 3001 Leuven, Belgium; quique.velascoserna@kuleuven.be; 5 Health Sciences Faculty, Centro Superior de Estudios Universitarios La Salle, Universidad Autónoma de Madrid, 28023 Madrid, Spain; mariagalanc9@gmail.com; 6 Motion in Brains Research Group, Centro Superior de Estudios Universitarios La Salle, Universidad Autónoma de Madrid, 28023 Madrid, Spain

**Keywords:** bilateral transcranial direct current stimulation (bi-tDCS), adverse effects, transcranial stimulation, patient safety, adverse effects’ temporal evolution, blinding protocol

## Abstract

(1) Background: Transcranial direct current stimulation (tDCS) is a safe intervention, only producing mild and transient adverse effects (AEs). However, there is no detailed analysis of the pattern of adverse effects in an application transferable to the clinic. Therefore, our objective is to describe the AEs produced by tDCS and its temporal evolution. (2) Methods: A total of 33 young volunteers were randomized into a tDCS or sham group. Participants performed a hand dexterity task while receiving the tDCS or sham intervention (20 min and 1 mA), for five consecutive days. AEs were assessed daily after each intervention and classified as somatosensory, pain, or other effects. (3) Results: The number of AEs was generally increased by tDCS intervention. Specifically, tDCS led to more frequent somatosensory discomfort, characterized by sensations like itching and tingling, alongside painful sensations such as burning, compared to the sham intervention. Additionally, certain adverse events, including neck and arm pain, as well as dizziness and blurry vision, were exclusive to the tDCS group. Interestingly, tDCS produced similar AEs across the days; meanwhile, the somatosensory AEs in the sham group showed a trend to decrease. (4) Conclusions: tDCS produces mild and temporary somatosensory and pain AEs during and across sessions. The different evolution of the AEs between the tDCS and sham protocol could unmask the blinding protocol most used in tDCS studies. Potential solutions for improving blinding protocols for future studies are discussed.

## 1. Introduction

In neuroscience research and clinical practice, non-invasive brain stimulation has become increasingly important. In this context, transcranial direct current stimulation (tDCS) stands out as one of the most investigated and developed modalities [1]. It involves the application of a weak direct current (1 or 2 mA) through two or more electrodes placed on the scalp for 5–30 min [2,3]. The potential clinical applications of tDCS have been thoroughly studied in a wide range of pathologies, including Parkinson’s and Alzheimer’s disease, stroke, multiple sclerosis, epilepsy, disorders of consciousness, tinnitus, depression, schizophrenia, addictions, and chronic pain [4].

Nowadays, it is well stablished that tDCS is clinically safe, very rarely producing any severe adverse effects (AEs), taking into consideration studies that ranged from 0.03 to 2.0 mA of current intensity and from 18 to 50 min of session duration [3,5,6,7,8]. Nevertheless, prior research reports the occurrence of mild and transient AEs associated with tDCS, such as: itching, tingling, discomfort, pain, burning sensation, skin irritation, headache, fatigue, sleepiness, nausea, insomnia, difficulty of concentration, and dizziness [7,8,9,10]. Additionally, a meta-analysis by Nikolin S. et al. [11] suggests a small heightened risk of AEs with increased exposure to tDCS.

Revision studies about this topic emphasize the lack of a systematic procedure for the collection and description of AEs which are frequently under-reported and improperly defined [7,11]. In addition, in the literature AEs are reported up to 102 sessions [5]; the main part of the studies predominantly assesses AEs following a single tDCS session [6]. This lack of continuity in the evaluation makes the knowledge less translational to clinical practice, where usually multiple sessions are involved. Moreover, most previous works evaluating tDCS safety consider participants during rest conditions, but in the clinical setting the intervention is used in combination with other approaches such as motor training and during repetitive sessions. Finally, to the best of our knowledge, there is existing literature investigating the temporal evolution of AEs across sessions [11] but neither the temporal evolution within nor between sessions in a clinical context is explored in detail. That exploration could contribute to the understanding of the AEs associated with tDCS.

The principal aim of this investigation is to describe the temporal dynamics of AEs produced by tDCS in a clinical context. For that purpose, we have assessed the intra- and inter-session temporal evolution of AEs, as well as their severity. Additionally, we have explored potential a priori factors that can predict the AEs’ emergence. To simulate clinical conditions, the tDCS intervention was applied simultaneously with a dexterity training task, a modality commonly employed in motor rehabilitation. The intervention was performed on healthy participants to avoid interactions between tDCS and pathological conditions, controlled with a sham procedure.

## 2. Materials and Methods

### 2.1. Study Design

This study employed a randomized, controlled, parallel, triple-blind clinical trial design. The allocation concealment was rigorously maintained throughout the entirety of the study, with the group assignments remaining undisclosed to all parties involved, including the recruiters, subjects, therapists, evaluators, and the statistician. The recruitment phase, conducted from April to June 2019, was primarily focused on volunteers associated with the CSEU La Salle—Faculty of Health Sciences in Madrid. Subsequently, participants were classified into the experimental (tDCS) or sham group (Sham tDCS). The allocation was executed through a permuted block randomization approach (GraphPad Software), ensuring a 1:1 allocation ratio. Importantly, classification of the participants was maintained for all the experimental procedure. This study is a secondary analysis of AEs reported during a tDCS clinical trial where motor learning was assessed.

### 2.2. Standard Protocol Approvals, Registrations, and Patient Consents

The study was approved by the GAE Clinical Research and Ethics Committee of the Hospital Infantil Universitario Niño Jesús (R-0022/18—approved 24 July 2018). It is important to note that the findings presented in this paper are a secondary analysis of the clinical trial registered as NCT03931512 (30 April 2019), the primary outcomes of which have been detailed in a prior study focused on measuring the impact of tDCS on motor learning [12]. First of all, participants signed an informed consent in accordance with the Declaration of Helsinki.

### 2.3. Participants

**Figure 1:**
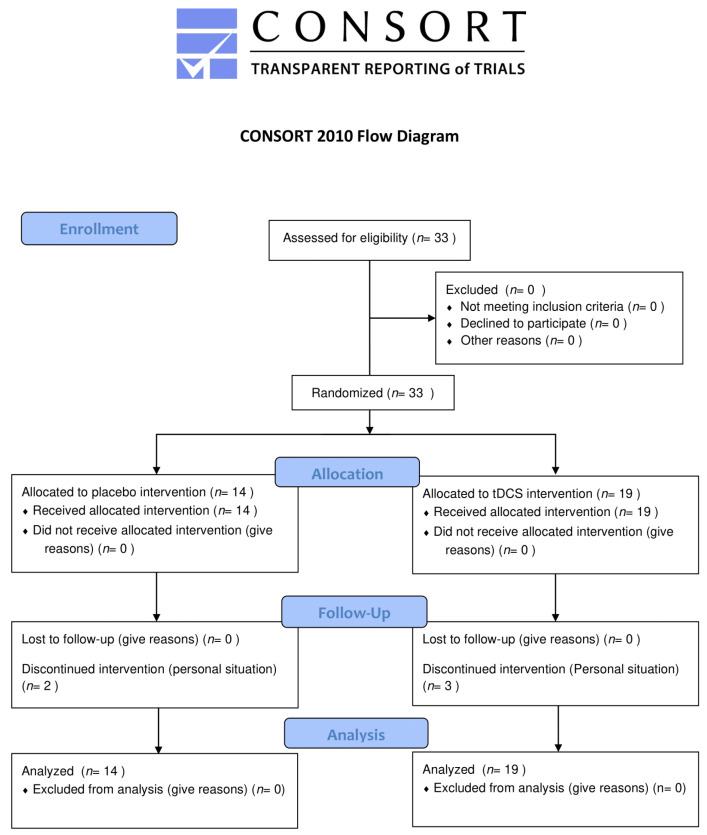
CONSORT 2010 flow diagram.

Thirty-three healthy and young (25.15 ± 4.2 years) volunteers (15 women) were recruited (Figure 1). The inclusion criteria involved an age range of 18−35 years old, recognizing the potential impact of age on performance and motor learning.

Exclusion criteria encompassed non-invasive brain stimulation (NIBS) considerations: presence of metal or skin lesions on the head, brain stimulation within the last 6 months, a family history of epilepsy or seizures, pacemaker or any cardiac involvement [3]. Additionally, individuals with pathologies that could affect the upper limb, an inability to understand or execute the assigned task, or those with a history of drug intake influencing cognition or engaging in harmful alcohol consumption (>40 g/day in women and >60 g/day in men) were excluded. Pregnancy was also considered an exclusion criterion.

### 2.4. Intervention

The intervention was applied with a multichannel wireless transcranial current stimulator (Starstim tCS^®^, Neuroelectrics^®^, Avinguda Tibidabo, 47 bis, 08035 Barcelona, Spain), programmed using NIC 2.0 software (Neuroelectrics^®^, Avinguda Tibidabo, 47 bis, 08035 Barcelona, Spain). The intervention consisted of five daily sessions, each lasting 20 min [4]. Concurrently, participants engaged in hand motor training with their dominant hand, constituting an online training paradigm [13,14]. This approach mirrors clinical conditions where tDCS is administered in combination with other therapies such as motor rehabilitation. For a comprehensive understanding of this protocol, see Flix-Díez L., et al., 2021 [12].

In terms of the tDCS montage, two saline-soaked circular sponge electrodes (25 cm^2^) were applied in a bi-hemispheric configuration, with the anode on the right primary motor cortex (M1) (C4) and the cathode on the left (M1) (C3). To ensure the accurate and reliable placement of electrodes on M1, a non-conductive, neoprene, adult-sized cap was employed (Neuroelectrics^®^ Neoprene Headcap), adhering to the international electrode placement system 10–20 [15]. The stimulator conducted an impedance check to guarantee optimal current flow, as indicated by the “contact quality” reading [16]. In cases where the impedance exceeded the acceptable threshold, additional saline was applied to facilitate an adjustment [17].

For the tDCS parametrization, the experimental group received a current intensity of 1 mA for 20 min, resulting in a current density of 0.04 mA/cm^2^. The protocol included an initial and final ramp of 10 s each, according to previous studies. Conversely, the sham group received a validated sham protocol [18]. The current was programmed to increase in intensity over 10 s, reaching a maximum of 1 mA, after which a 20 s down ramp was initiated until the current reached 0. Consequently, participants in the control group were subjected to a mere 30 s of stimulation at the beginning of each session. The authors of the protocol proposed that the minimal current perception evoked at the beginning of the sham protocol is sufficient to mask the intervention [3,18]. Additionally, the blinding procedure for the therapist, assessor, and statistician is described in the Appendix A.

### 2.5. Measurement Protocol

The measurement protocol was consistently executed by the same researcher. Participants completed a questionnaire detailing sociodemographic characteristics: age, gender, level of education, personal and family history, and drug intake. The Edinburgh Handedness Questionnaire was employed to determine their dominant hand [19].

After each intervention, AEs were assessed. Participants were queried about their perceptions during the first minute (when both groups received the current application), during the remaining intervention (only the tDCS group received the current application), and once the intervention finished (both groups without current application). This temporal division was established in consideration of the expected appearance times of AEs [5]. In addition, participants were encouraged to communicate with the research group if they experience any additional AEs in the following days, although such instances did not occur.

The primary variables extracted were the percentage of participants reporting AEs and the number of AEs reported by each subject. A categorization of AEs into somatosensory, pain, or other effects facilitated subsequent analysis. Dropouts were recorded, which could be potentially interpreted as an additional indirect indication of AEs. This possibility is particularly relevant in scenarios where the tDCS group exhibits a higher dropout rate compared to the sham group, and such dropout rates are found to be correlated with certain AEs.

To gauge the positive and negative moods of the participants, we used the Spanish validated self-reported questionnaire, Positive Affect Negative Affect Schedule (PANAS), after each intervention [20].

Additionally, to explored potential a priori factors that could predict the AEs’ emergence, we decided to assess physical activity and sleep quality using the International Physical Activity Questionnaire (IPAQ) (Long version: USA Spanish version 3/2003) [21] and the Pittsburgh Sleep Quality Index (PSQI) [22]. On one hand, the IPAQ presents questions related to physical activities performed in the different domains of daily life. The total score of the IPAQ is reported in MET minutes/week, determining the level of physical activity (low, moderate, or high). On the other hand, the PSQI is one of the most recommended instruments to assess sleep quality and it consists of 19 items that analyze different determinants of sleep quality grouped into seven components: quality, latency, duration, efficiency, sleep disturbances, use of medication for sleep, and diurnal dysfunction [22]. The total score of the PSQI is between 0 and 21, with a higher score denoting worse sleep quality. Subjects with more than 5 points are considered poor sleepers. Due to the retrospective nature of both instruments, those questionnaires were retrospectively fill out by participants after completing the 5-day intervention.

Lastly, somatosensory thresholds were recorded to assess potential correlations between somatosensory and pain processing and the reported AEs. Mechanical detection and pressure pain thresholds over the thenar eminence were determined prior to any intervention, utilizing The Touch-Test™ Sensory Evaluators (Semmes–Weinstein Monofilaments) [23] and a handheld digital algometer (FDX-25 Wagner Instruments, Greenwich, CT) [24], respectively.

### 2.6. Sample Size Calculation

The sample size calculation for this study was previously documented in Flix and Delicado-Miralles et al. 2021 [12]. The primary outcome of the clinical trial, specifically the change in manual dexterity of the dominant hand, was used to determine the efficacy of the treatment. With 5% alpha and 20% beta errors and an a priori effect size of 1.3, inferred from Waters et al. [25], the initial sample size calculation resulted in a requirement of 22 subjects (11 in each group). However, to account for possible dropouts, an additional 25–30% of subjects (+6) were included, reaching a recruited sample of 28 subjects. Given this sample size, we estimated the statistical power for the analysis of AEs reported here establishing a 5% alpha error, resulting in 0.85 for the variable “percentage of subjects reporting somatosensory AEs during the session”, which is a variable often reported in tDCS AEs studies.

### 2.7. Statistical Analysis

Statistical analyses were performed using IBM SPSS Statistics (Version 25.0). The normality of variables was assessed through the Shapiro–Wilk test and histogram distributions. Depending on normality assumptions, parametric and non-parametric tests were used. Categorical variables were expressed as a frequency or percentage and the group differences were compared with the χ^2^ test. Quantitative variables were presented as mean and standard deviation or median and interquartile range.

To examine variations in reported AEs over time, we used either repeated measurements ANOVA or binary logistic regression models, depending on the variable type, quantitative or categorical, respectively. Cumulative incidence of AEs or dropouts was depicted using the Kaplan–Meier Method and Log-Rank Test. A binary logistic regression model was used to explore a priori factors predicting the appearance of AEs. For analytical clarity, AEs were categorized into three groups: somatosensory (itching, heat, and tingling), pain (pricking pain, burning pain, headache, arm pain, and neck pain) and other (fatigue, dizziness, and blurry vision). Dropouts were included in the analysis (intention to treat analysis). The statistical significance threshold was set at *p* < 0.05.

## 3. Results

Five subjects did not complete the five treatment sessions, citing personal matters unrelated to the study as the reason for their non-compliance (Figure 1). These dropouts were included in the analysis, employing an intention-to-treat approach. Descriptive data for all subjects, inclusive of dropouts, are presented in Table 1. There were no significant differences in variables between the groups, except for the age of the subjects, which was marginally higher in the tDCS group.

### 3.1. General AEs Analysis

**Figure 2:**
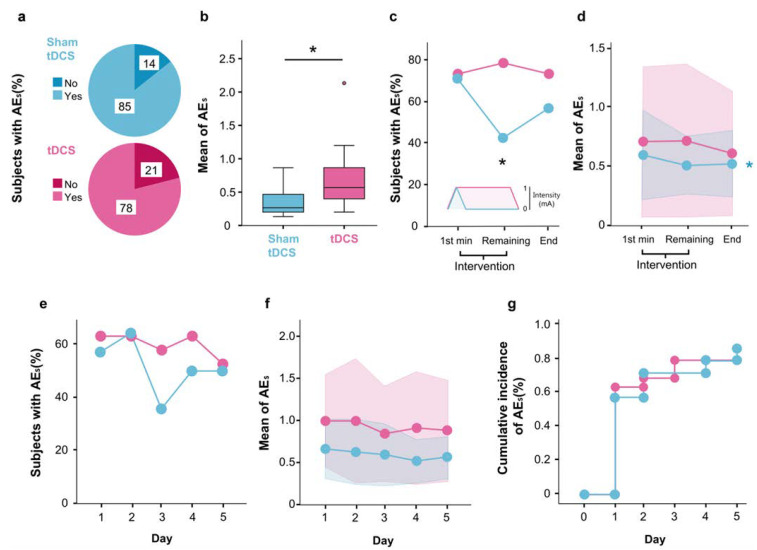
tDCS effects over the total AEs reported by subjects. (**a**) Percentage of subjects that scored any type of AEs. (**b**) Mean of total AEs scored by subjects per session. (**c**) Percentage of subjects that scored any type of AEs at the different time points of a single session. (**d**) Mean of total AEs scored during at the different time point of a single session. (**e**–**f**) The same metrics described in “c” and “d” across the days. (**g**) Cumulative incidence of subjects reporting AEs across the days. Signification threshold was *p* < 0.05 and was indicated by one asterisk (*), indicating if the comparisons were within (colored with the color of the group) or between groups (in black). Outliers are represented by dots.

The occurrence of AEs during the intervention period was reported by 81.82% of participants in both the tDCS and sham groups (Figure 2a). However, the tDCS group exhibited a greater incidence of AEs per participant in comparison to the sham group (0.36 ± 0.15 mean and SD difference, *p* = 0.026, Cohen’s d = 0.95) (Figure 2b). Thus, while the likelihood of experiencing at least one AE was similar in both groups, participants who received tDCS tended to report a greater number of AEs.

A comparison of the intra-session time evolution of AEs appearance between the two groups revealed that, during the first minute, when both groups received the current, the percentage of participants experiencing AEs between groups was nearly identical (73.7% for tDCS and 71.4% for the Sham group). Conversely, during the remaining intervention period (only the tDCS group receiving the current), the tDCS group exhibited a higher percentage of subjects reporting AEs compared to the sham group (78.9% vs. 42.9%, *p* = 0.033) (Figure 2c). The number of AEs reported by each participant decreased throughout the session in the sham group (*p* = 0.024, η_p_^2^ = 0.339), but not in the tDCS group (*p* = 0.388, η_p_^2^ = 0.070) (Figure 2d). These results indicate that the AEs could be associated with the transcranial current.

For the inter-session evolution of AEs, the percentage of participants reporting AEs was similar between groups (*p* = 0.713, Nagelkerke R^2^ = 0.17) (Figure 2e). The same is observed for the quantity of AEs observed (*p* = 0.984, η_p_^2^ = 0.023) (Figure 2f). A survival analysis revealed that the cumulative incidence of AEs reported by participants across the five treatment sessions was comparable between the two groups (*p* = 0.977, Log-Rank (Mantel–Cox) Chi^2^ = 0.047) (Figure 2g).

### 3.2. Type-Specific AEs Analysis

To provide a more comprehensive analysis, all reported AEs are detailed in Table 2. Thereafter, we have classified AEs in three main types: somatosensory, pain, and other.

**Figure 3:**
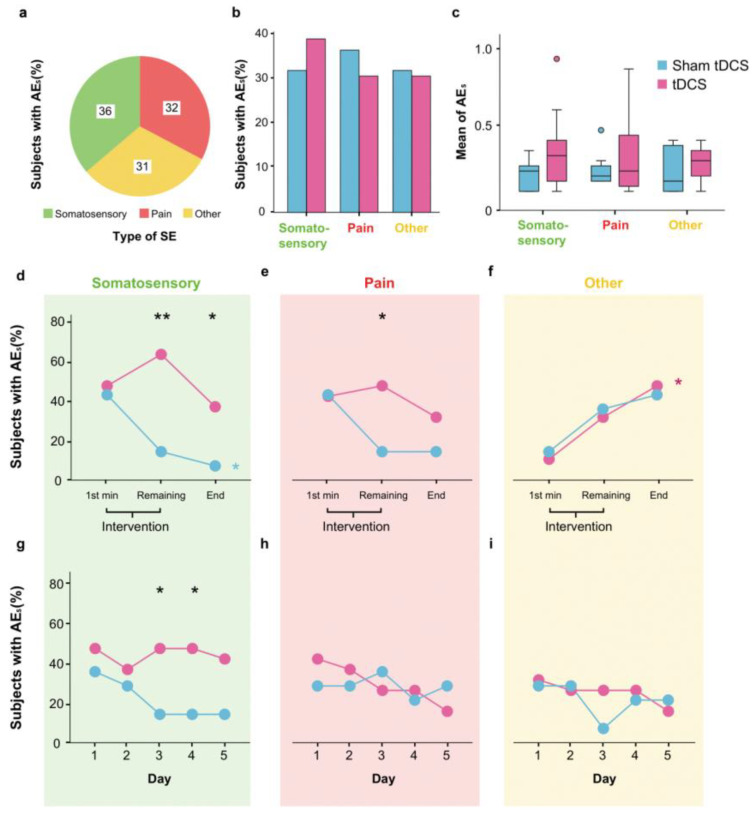
tDCS effects over the three types of AEs. (**a**) The frequency of participants reporting the three different types of AEs combining and (**b**) comparing both groups. (**c**) Average AEs scored per subject. (**d**–**f**) Percentage of subjects reporting each type of AE during a single session (**g**–**i**) and along 5 daily sessions. The signification thresholds were *p* < 0.05 and *p* < 0.01, indicated by one or two asterisks (*), respectively. Color of the asterisk indicates if the comparisons were within (colored with the color of the group) or between groups (in black). Outliers are represented by dots.

The probability of experiencing somatosensory, pain, and other AEs was similar (*p* = 0.747) (Figure 3a), and this pattern was consistent across the tDCS and sham groups (*p* = 0.947, 0.095 and 0.142, respectively) (Figure 3b). Indeed, the result was the same for the quantity of somatosensory, pain, and other AEs observed between groups (*p* = 0.173, 0.768 and 0.747, respectively) (Figure 3c).

We also analyzed the intra-session and inter-session evolution of each AE type. Intra-session analysis of somatosensory AEs revealed group differences during the intervention (63.2% for tDCS vs. 14.3% for sham, *p* = 0.005) and after the intervention (36.8% and 7.1%, *p* = 0.049), excluding the first minute (47.4% and 42.9%, *p* = 0.797) (Figure 3d). As we observed in the intra-session total AEs, the somatosensory AEs tended to decrease in the sham group during the session (from 42.9% to 7.1%, *p* = 0.051), but not in the tDCS group (from 47.4% to 36.8%, *p* = 0.263). Pain AEs showed a similar tendency to somatosensory AEs (Figure 3e), being similar in first minute (42.1% and 42.9%, *p* = 0.966), and different during the rest of the intervention (47.4% and 14.3%, *p* = 0.046) and after the intervention (31.6% and 14.3%, *p* = 0.252). Other AEs showed a different pattern, tending to increase in both groups across session, without differences between groups (Figure 3f). Intra-session data about the percentage of participants experiencing different AEs are summarized in Table 3.

Inter-session analysis demonstrated that the somatosensory, pain, and other AEs did not change in the sham group (*p* = 0.96, 0.852, and 0.536; Nagelkerke R^2^ = 0.06, 0.001, and 0.008, respectively) or the tDCS group (*p* = 0.999, 0.054, and 0.315; Nagelkerke R^2^ = 0.001, 0.054, and 0.016, respectively). However, we found a reduction in the percentage of participants with somatosensory AEs in the sham group compared to the tDCS group on the third and fourth day (*p* = 0.046) (Figure 3g). On day 5, although a similar tendency in somatosensory AEs was observed, it did not reach significance (*p* = 0.085). Specifically, we have observed that this discrepancy is attributable to a single subject who altered their report on the fifth day of the tDCS group, with all the other participants remaining consistent.

### 3.3. Other Variables Analysis

**Figure 4:**
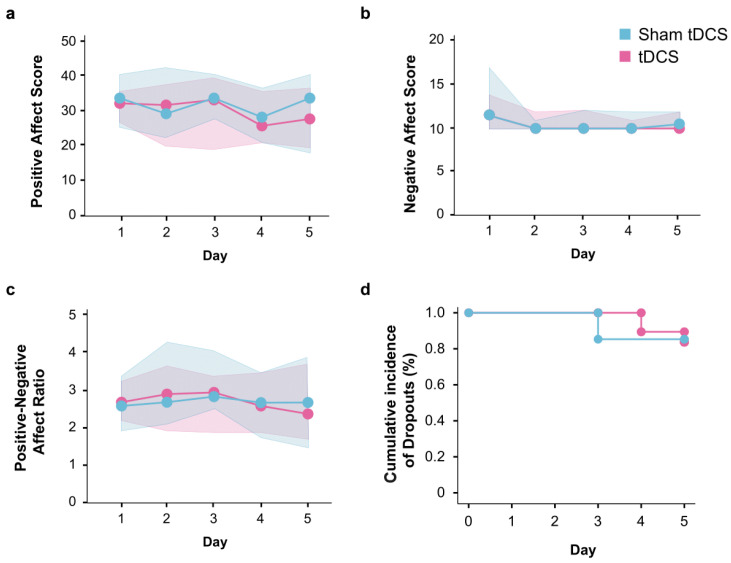
tDCS effects over the PANAS score and dropout analysis across days. Positive (**a**), negative (**b**), and the ratio (**c**) mood scale (PANAS) scores compared between groups. (**d**) Incidence of dropouts compared between groups.

Concerning mood-related effects over the intervention days, no difference was observed in Positive, Negative, or Positive–Negative Affect Score Ratio from the PANAS questionnaire between the sham (*p* = 0.264, 0.16, and 0.361, respectively) and tDCS groups (*p* = 0.318, 0.005, and 0.318) (Figure 4a-c). Dropout analysis showed similar rates between groups, with a total cumulative incidence of 15%, and all dropouts occurring between days 3 and 4 (Figure 4d).

Finally, we tried to predict AEs incidence using the a priori variables recorded from participants. The binary logistic regression model, which included age, sex, PANAS, physical activity, sleep, and somatosensory thresholds, was not significant (*p* = 0.579, Nagelkerke R^2^ = 0.001), indicating that none of these factors serve as a predictor for AEs appearance.

## 4. Discussion

The main findings of this study showed a different temporal evolution of the different types of tDCS AEs, intra-session and inter-session, in order to establish the relation between tDCS and AEs in a setting similar to clinical applications. To achieve this goal, we have studied the AEs’ temporal pattern evoked during a motor rehabilitation approach: a repetitive application combined with a motor task across five consecutive days. Our main finding was the close association between specific AEs, such as itching, neck and head pain, blurry vision, and dizziness, and tDCS intervention. Conversely, other AEs typically attributed to tDCS, such as fatigue or headache, were not related to the transcranial current. Additionally, the observed temporal evolution of somatosensory AEs (intra-session and the inter-session) between groups. Specifically, the observed difference between the groups in the temporal evolution of these AEs was found to decrease at the sham intervention but to remain stable at the tDCS intervention, which has significance for the development of effective blinding protocols in this field.

As it was expected, there was a close association between certain AEs, such as itching, neck and head pain, blurry vision and dizziness, and tDCS intervention, while other AEs typically attributed to tDCS, such as fatigue or headache, were not related to the transcranial current. As a general analysis, we found that tDCS application did not increase the percentage of participants with AEs but increased the overall number of AEs scored by participants compared to the sham intervention. Itching, tingling, burning, and fatigue were the most frequently reported AEs, aligning with findings from other studies [3,5,11,20,26]. Furthermore, we identified other less common AEs such as pricking neck and arm pain, dizziness, or blurry vision. Notably, blurry vision, dizziness, and neck and arm pain were exclusive to the tDCS group, suggesting a potential link to the brain stimulation itself. According to the Common Terminology Criteria for Adverse Events [27], all AEs reported in this study would be classified as mild adverse events (MAEs—grade 1: symptoms not requiring medical care).

Skin damage, frequently reported in the literature [28], was not observed in our participants. This may be attributed to the use of saline solution as an interface between the electrode sponges and the scalp, coupled with the procedure to ensure consistent electrode–skin contact pressure. Although the intensity applied was low (1 mA), the evidence suggests that skin lesions can also occur at this intensity of stimulation (1 mA/0.029 mA/cm^2^) [5]. This underscores the significance of protocol adherence to prevent skin damage.

Exploring the intra-session evolution of adverse events (AEs), we observed a similar pattern for somatosensory and pain types. For intra-session evolution, during the first minute, around 50% of the participants in both groups reported somatosensory and pain AEs. However, throughout and after the intervention, the tDCS group reported more AEs, particularly itching. This temporal variation aligns with the sham procedure, applying 30 s of current at the beginning to create tingling or itching sensations, potentially masking the actual treatment [18]. Pain AEs like arm and neck pain were exclusive to the tDCS group, establishing a clear association between current application and somatosensory and pain AEs.

Concerning the nature of the pathophysiology of the somatosensory AEs, we remark that those are circumscribed to the area of stimulation and closely related to the current stimulation. The main explanation for those sensations is that they seem not to be a consequence of the central nervous system stimulation but the somatosensory peripheral nerves innervating the skin. This suggestion is supported by some studies that have found that the use of a peripheral anesthetic would reduce those sensations [29,30]. However, we cannot discard that some sensations could potentially be a consequence of the central nervous system stimulation, as M1 is implicated in pain processing [31,32] and has a close relation with somatosensory cortex areas [33,34]. Regarding the intra-session evolution of other AEs (dizziness, blurry vision, and fatigue), the tDCS group showed a significant increase throughout the session, but without differences between groups.

Analyzing the inter-session AEs evolution, we found that somatosensory AEs were the unique AE that varied across the five days, decreasing consistently in the sham group. These results point in the same direction as those of Paneri B. et al. [35], where a decrease in tingling sensations was also observed over the first 2–3 sessions. We hypothesize that the subjects in the sham group were becoming accommodated to the procedure, reducing their fear and expectancy of adverse effects, key contributors to the nocebo effect [36]: a psychological phenomenon which makes AEs appear more probable [37]. Since our subjects were inexperienced with non-invasive brain stimulation, this adaptation is plausible.

The detection of AEs in the tDCS group compared to the sham group raises concerns about the blinding integrity of our study. This is inherent to the technique and study type, and thus calls into question the efficacy of blinding protocols such as the Gandiga sham protocol [18]. The protocol employs brief stimulation to induce perceptions, aiming to prevent subjects from differentiating between the tDCS and sham through the resulting itch, maintaining blinding. However, our findings reveal a decrease in somatosensory and pain AEs, specifically in the sham group after current disconnection for the rest of the session. This differs from the original validation paper, which quantified the total number of AEs throughout the session. This approach failed to capture the distinct intra-session temporal evolution in both groups [18]. Despite the original report claiming no subject could distinguish between treatments, the induction of different somatosensory perceptions could impact subsequent results.

Brain stimulation induces AEs and directly compromises blinding. As a potential solution, we propose removing somatosensory sensations over the skin, such as by using a topical anesthesia under the electrode, or inducing them in both groups with an external source unrelated to the current, like a capsaicin or lidocaine cream. In addition, it should be taken into consideration that some part of the tDCS effect could be mediated by this peripheral stimulation [29,38,39].

Our a priori model failed to predict AEs. Thus, the introduced variables (age, sex, PANAS, physical activity, sleep, and somatosensory thresholds) are unrelated to the appearance of AEs, at least within the range explored in this study.

In terms of study limitations, we highlight that the participants were healthy and young, which may limit the external validity of our findings to a clinical context. Moreover, the retrospective assessment of AEs at the end of the intervention, while aimed at maintaining participant focus, might lead to underestimating AEs reporting. Skin redness, a potential side effect, was not specifically evaluated in this study. For future investigations, delving into the time evolution of AEs in pathological conditions and diverse clinical settings would be insightful. Another important limitation of the present work is only evaluating the presence of AEs but not the severity of each AE. However, we can robustly confirm that all AEs observed during this study can be classify as mild based on duration (restricted to the intervention or 5 min after the intervention) and the observation during the informal conversation with each patient after each intervention. Additionally, in this experimental paradigm in which we have tested a combination of tDCS (real or sham) and a motor task, we cannot completely exclude the potential influence of the motor task on the observed AEs. However, the somatosensory AEs’ close correlation with current administration tends to decrease over time in the sham group, suggesting minimal influence from manual tasks. Furthermore, incorporating objective physiological measures, such as fatigue-related variables or skin impedance, instead of solely relying on self-reported AEs, could provide valuable insights into the biological underpinnings of AEs. We also identify as a limitation the retrospective collection of the IPAQ and PSQI questionnaires. They could be influenced by treatment conditions. Finally, we must remark that we applied a current intensity of 1 mA, which is low compared to common protocols. This could conditionate the external validity of our results to other protocols that apply higher intensities of stimulation (>1 mA). It can be reasonably assumed that the probability of experiencing adverse effects (AEs) will increase in direct proportion to the intensity of stimulation.

## 5. Conclusions

The main conclusion of this work is that tDCS produces mild and temporary somatosensory and pain AEs during and across sessions. Specifically, the tDCS-related AEs were mainly itching and pricking and burning pain. Surprisingly, blurry vision, dizziness, and neck and arm pain were AEs exclusive to the tDCS group. In addition, somatosensory AEs produced in the sham protocol decreased during a single session and across the days, which could compromise the blinding protocols of the studies. No physical, demographic, or affective variable in this study constituted a predictor for AEs. Finally, the temporal analysis of AEs provides us valuable information for a deeper understanding of the AEs induced by tDCS, which appears to be safe for use during five days of motor dexterity training in a healthy population.

## Figures and Tables

**Table 1:** Descriptive analysis of sociodemographic data. To evaluate the homogeneity of the different variables between groups Student’s t was used for quantitative variables and Pearson’s chi-squared test for categorical variables.

Descriptive Variables		Sham Group (*n* = 14)	tDCS Group (*n* = 19)	*p*-Value
Quantitative (mean ± standard deviation)				
Age		23 ± 1.2	26.7 ± 4.9	0.001
Sleep Quality (Pitsburg)		6.4 ± 3.2	4.6 ± 2.3	0.12
Physical activity (IPAQ)		7963 ± 5875	8794 ± 5359	0.71
Qualitative (number of participants (percentage))				
Gender	Man	6/14 (42.9)	12/19 (63.2)	0.21
	Woman	8/14 (57.1)	7/19 (36.8)	
Laterality	Left-handed	1/14 (7.1)	4/19 (21.1)	0.27
	Right-handed	13/14 (92.9)	15/19 (78.9)	
Education	Higher	1/14 (7.1)	4/19 (21.1)	0.27
	High school	13/14 (92.9)	15/19 (78.9)	

**Table 2:** Specific AEs scored by subjects (number of participants that reported AEs (percentage)). The bold font indicates AEs that appeared only in the tDCS group. The signification *p* < 0.05 was indicated by one asterisk (*).

AE Classification	Specific AE	Sham Group (*n* = 14)	tDCS Group (*n* = 19)	Total (*n* = 33)
Somatosensory AE	Itching *	2 (14.3)	10 (52.6)	12 (36.4)
	Heat	1 (7.1)	1 (5.3)	2 (6.1)
	Tingling	4 (28.6)	4 (21.1)	8 (24.2)
Pain AE	Pricking pain	3 (21.4)	5 (26.3)	8 (24.2)
	Burning pain	4 (28.6)	3 (15.8)	7 (21.2)
	Headache	2 (14.3)	2 (10.5)	4 (12.1)
	Neck Pain	**0**	**2 (10.5)**	**2 (6.1)**
	Arm Pain	**0**	**4 (21.1)**	**4 (12.1)**
Other AE	Fatigue	7 (50)	7 (36.8)	14 (42.4)
	Dizziness	**0**	**3 (15.8)**	**3 (9.1)**
	Blurry vision	**0**	**2 (10.5)**	**2 (6.1)**

**Table 3:** Specific AEs scored intra-session (number of participants that reported AEs (percentage)). The bold font indicates AEs appearing only in the tDCS group.

AEs Classification Specific		Sham Group (*n* = 14)			tDCS Group (*n* = 19)		
		1st min	Remaining	End	1st min	Remaining	End
Somatosensory	Itching	2 (14.3)	0	0	6 (31.6)	8 (42.1)	5 (26.3)
	Heat	1 (7.1)	1 (7.1)	0	0	1 (5.3)	0
	Tingling	3 (21.4)	1 (7.1)	1 (7.1)	4 (21.1)	3 (15.8)	2 (10.5)
Pain	Pricking pain	3 (21.4)	0	0	4 (21.1)	5 (26.3)	0
	Burning pain	4 (28.6)	0	0	3 (15.8)	2 (10.5)	1 (5.3)
	Headache	0	2 (14.3)	2 (14.3)	0	1 (5.3)	2 (10.5)
	Neck Pain	**0**	**0**	**0**	**0**	**1 (5.3)**	**1 (5.3)**
	Arm Pain	**0**	**0**	**0**	**3 (15.6)**	**3 (15.6)**	**2 (10.5)**
Other	Fatigue	2(14.3)	5 (35.7)	6 (42.9)	1 (5.3)	4 (21.1)	7 (36.8)
	Dizziness	**0**	**0**	**0**	**1 (5.3)**	**2 (10.5)**	**2 (10.5)**
	Blurry vision	**0**	**0**	**0**	**1 (5.3)**	**2 (10.5)**	**0**

## Data Availability

The datasets generated in this study are publicly available in the OSF repository: https://osf.io/gszc5/ (DOI 10.17605/OSF.IO/GSZC5).

## Appendix A. Blinding Procedure

Participant allocation was concealed to everyone who participated in the experiment: participants, therapist, assessor, and statistician. The blinding procedure started with the intervention codification performed by the therapist and the pre-programming of both interventions in the device under the name of 0 and 1 protocols (tDCS = 0 and sham tDCS = 1). Then, the assessor, being blinded, performed a second codification (0 = B and 1 = A), allocated the participants, and changed the names of the device programs (Protocol A and B). In this way, neither of them knew which protocol was A or B. To preserve blinding, other aspects of the tDCS device were customized. During the intervention, the device did not show the current intensity administered, but only a categorical indication of the “quality of contact” from the electrodes (optimal, moderate, or bad), reducing the risk of unmasking [40]. Finally, for allocation and blinding, participants were instructed not to discuss the intervention with the evaluator or the therapist. The research staff member who analyzed the data of the study was also blinded. The blinding was maintained until the study was completed and the dataset was blocked.
